# Single-Chain Fragment Variable Passive Immunotherapies for Neurodegenerative Diseases

**DOI:** 10.3390/ijms140919109

**Published:** 2013-09-17

**Authors:** Liang Huang, Xiaomin Su, Howard J. Federoff

**Affiliations:** 1 Department of Neuroscience, Georgetown University Medical Center, Washington, DC 20057, USA; E-Mails: lh629@georgetown.edu (L.H.); xs37@georgetown.edu (X.S.); 2 Department of Neurology, Georgetown University Medical Center, Washington, DC 20057, USA

**Keywords:** scFv, immunotherapy, Prion disease, Alzheimer’s disease, Parkinson’s disease, Huntington’s disease, PrP, Aβ, SNCA, Htt

## Abstract

Accumulation of misfolded proteins has been implicated in a variety of neurodegenerative diseases including prion diseases, Alzheimer’s disease (AD), Parkinson’s disease (PD), and Huntington’s disease (HD). In the past decade, single-chain fragment variable (scFv) -based immunotherapies have been developed to target abnormal proteins or various forms of protein aggregates including Aβ, SNCA, Htt, and PrP proteins. The scFvs are produced by fusing the variable regions of the antibody heavy and light chains, creating a much smaller protein with unaltered specificity. Because of its small size and relative ease of production, scFvs are promising diagnostic and therapeutic reagents for protein misfolded diseases. Studies have demonstrated the efficacy and safety of scFvs in preventing amyloid protein aggregation in preclinical models. Herein, we discuss recent developments of these immunotherapeutics. We review efforts of our group and others using scFv in neurodegenerative disease models. We illustrate the advantages of scFvs, including engineering to enhance misfolded conformer specificity and subcellular targeting to optimize therapeutic action.

## 1. Introduction

Correct folding of proteins is essential for their functions [1]. Abnormal protein folding could lead to inaction, degradation, incorrect trafficking, and aggregation of proteins. In the latter cases, aggregation of certain proteins, such as Prion protein (PrP), Huntingtin (Htt), amyloid β (Aβ), α-Synuclein (SNCA) and Cu/Zn superoxide dismutase (SOD1), are mechanistically linked to neurological disorders such as prion diseases, Alzheimer’s disease (AD), Parkinson’s disease (PD), Huntington’s disease (HD) and amyotrophic lateral sclerosis (ALS) [2–8].

**Figure 1:**
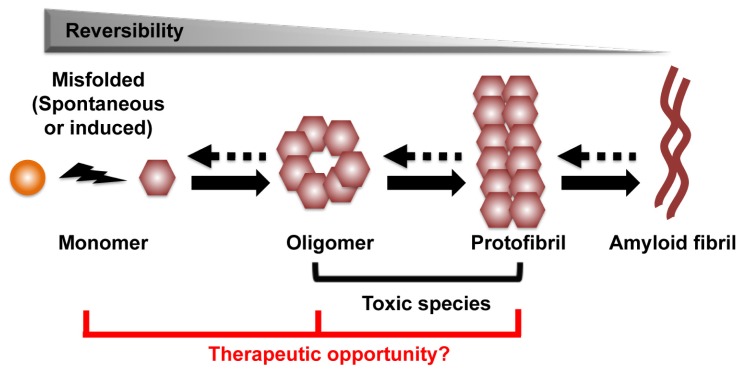
The process of amyloid formation. The first step of the process can be a conversion from the native α-helix rich soluble cellular protein into the pathogenic β-sheet rich isoforms, in which they are able to self-assemble through a variety of subsequent nucleation and growth steps to form oligomer, protofibril, and eventually amyloid fibril aggregates. Oligomer and protofibril are putative toxic species that drive neuronal dysfunction. We hypothesize the therapeutic targets are monomer, oligomer and protofibril.

The process that leads to the formation of protein aggregates is a reversible heterogeneous multistep reaction. Amyloid formation can start from conversion of the native α-helix rich soluble cellular protein into the putatively pathogenic β-sheet rich isoforms, which are able to self-assemble through nucleation and monomer additions to form oligomers, protofibrils, and eventually amyloid fibril aggregates (Figure 1) [9,10]. Other aggregation pathways are also possible. Structural analysis and computer modeling suggested that amyloid aggregate can initiate directly from misfolded protein fragments, and that fibrils can act as seeds for protein aggregation [11,12]. An accumulated body of evidence indicates that the amyloid aggregates might not be responsible for the primary neurotoxic effects of these disorders. Rather, oligomers and protofibrils are presumptively the toxic species that drive neuronal dysfunction and death [12–15]. Thus, strategies to prevent oligomer/protofibril formation, promote their elimination or inhibit their toxic activity may be therapeutic. As an increase in monomer concentration enhances oligomer formation, approaches to reduce monomers also appear to be a viable therapeutic approach (Figure 1) [14].

Active or passive immunotherapies directed against the disease-associated proteins have been prosecuted in preclinical and clinical studies [16–22]. Advancements in antibody technologies offer the scientific and clinical communities with better tools for diagnosis and treatment of neurological disease related to misfolded protein targets.

**Figure 2:**
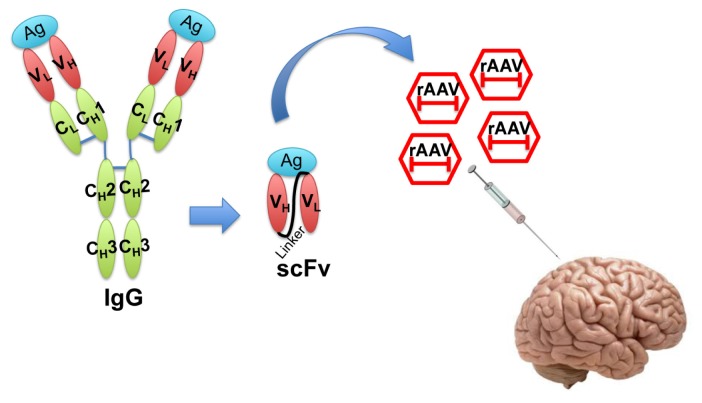
Illustration of single-chain fragment variable (scFv) structure and recombinant adeno-associated virus (rAAV) delivery of scFv. Engineered scFv lacking the constant regions of IgG is a much smaller molecule that still retains the antigen binding affinity. scFvs can be packaged into rAAV and efficiently delivered *in vivo* for therapeutic purposes. Ag: Antigen; V: variable region; C: constant region; L: light chain; H: heavy chain.

In the recent decade, single-chain fragment variable (scFv) antibodies have emerged as an option for passive immunotherapy [19]. The scFvs are generated by fusing the V_H_ and V_L_ fragments of an IgG with a suitable linker peptide [23]. The resulting molecule is relatively small and lacks the constant regions of the antibody, yet retains antigen-binding specificity. scFvs can be packaged in small viral vectors such as recombinant adeno-associated virus (rAAV) for direct injection into the central nervous system (Figure 2).

Compared with chimeric or humanized antibodies, scFvs have several advantages. First of all, they are among the smallest antibodies that still retain antigen-binding specificity although affinity can be reduced. Through intracerebral injection, intranasal administration and viral transduction, scFvs can be delivered and spread throughout the brain [17,24–26]. scFvs have characteristics that may improve their function compared to conventional murine monoclonal antibody (mAb), Fab or (Fab’)_2_ fragments. scFvs have little immunogenicity and because of the absence of a constant region, they do not fix complement. The small size of the scFvs yield better tissue penetration but they tend to have shorter half-lives. Furthermore, since scFvs do not require glycosylation, they can be produced in a bacterial expression system thus allowing production at a substantial scale [27].

To develop effective scFv immunotherapy for the neurodegenerative diseases associated with protein misfolding, two questions should be addressed: (1) Is there a specific conformation of misfolded protein target that is causally linked to disease mechanism? (2) Does the specific misfolded and pathogenic target produce injury in an extracellular, intracellular or subcellular compartment? Herein, we review studies on scFv mediated immunotherapeutic approaches for several neurodegenerative diseases caused by protein misfolding.

## 2. scFv Therapy in Prion Diseases

Prion diseases or transmissible spongiform encephalopathies (TSE) are a group of diverse transmissible, fatal diseases that feature the conversion of normal cellular expressed prion proteins (PrP^C^) into a pathological conformation (PrP^Sc^). The human forms of the disease include fatal familial insomnia, kuru, Creutzfeldt-Jakob disease, and Gerstmann-Straussler-Scheinker disease [9,28]. Antibodies against PrP^c^ could, indirectly, promote the clearance of the PrP^sc^, prevent the conversion from PrP^c^ into PrP^sc^ [26], inhibit the transport of PrP^c^ to the cell surface [29], or enhance the degradation of cellular PrP^c^ [26]. Generally, PrP^c^ is considered as a monomeric isoform of the prion protein rich in α-helical structure and thus sensitive to digestion by proteinase K; while the PrP^sc^ is a higher order form rich in β-pleated sheet structure and is resistant to proteinase K digestion [30,31].

There are at least 20 studies that used scFv to target prion proteins [24–26,29,32–48]. Many of the scFvs are capable of inhibiting PrP aggregation and reducing PrP^sc^-related cellular toxicity. Earlier attempts were made by Leclerc *et al.* and Flego *et al.* using phage display libraries expressing human scFvs to isolate anti-PrP antibody fragments [32,35]. scFvs can also be engineered based on the coding sequences of the variable regions of established murine monoclonal antibodies against prion proteins [25,33,49,50].

**Figure 3:**
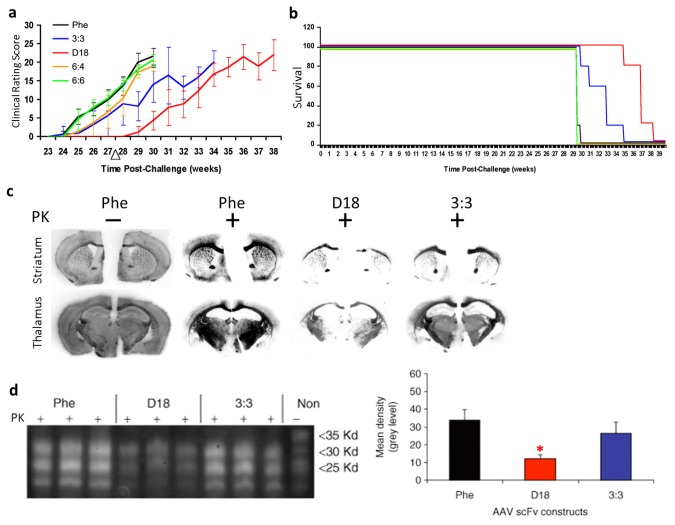
Outcomes of recombinant adeno-associated virus (rAAV) delivered single-chain fragment variable (scFv) therapy for prion disease in transmissible spongiform encephalopathies (TSE) mouse model. (**a**) Clinical rating data show that the disease onset was delayed in mice administered rAAV2-scFvD18; (**b**) Gehan-Wilcoxon test data show that the incubation period of prion disease was delayed in mice treated with scFv3:3 and scFvD18; (**c**) Histoblot analyses of brain sections show decreased levels of proteinase K-resistant PrP^sc^ in scFvD18 and scFv3:3 treated mice; (**d**) Immunoblots of brain homogenates (left panel) show a decreased level of proteinase K-resistant PrP^sc^ in scFvD18 and scFv3:3 treated mice. Quantitative measurements (right panel) of the immunoblots indicate that the decrease in scFvD18 group is significant when compared with the Phe control (marked with “*”, *p* < 0.004, *t*-test). PK: proteinase K; “−”: no PK digestion; “+”: PK digested. Figures reproduced from Wuertzer *et al.*, 2008 [39].

Our group developed passive immunomodulation with rAAV2 delivered anti-PrP^c^ scFv in a mouse model of prion disease [39]. We first identified three novel anti-PrP^c^ scFvs (3:3, 6:4, and 6:6) by screening the pAPII_6_ phagemid library. The scFv version of D18, a previously characterized anti-PrP Fab [13], was also generated. The four rAAV vectors expressing the anti-PrP scFvs, respectively, and a control scFv against an irrelevant antigen, phenobarbital (Phe), were administered intracerebrally into mouse prion model. All scFvs were engineered to be secreted efficiently with the aid of a murine immunoglobulin κ-secretory signal. Our results showed that treatment with anti-PrP^c^ rAAVscFv delayed the onset of the prion disease in a mouse model. Clinical signs of prion disease in mice treated with scFvD18 appeared about 1 month later than all other groups, as evaluated by clinical rating scores (Figure 3a). The incubation period of the disease was significantly delayed in mice treated with scFv3:3 and scFvD18 (Figure 3b). The secreted anti-PrP^c^ scFvs delayed the formation of proteinase K-resistant PrP^sc^ and thus significantly reduced the PrP^sc^ burden (Figure 3c,d). In summary, our work has demonstrated the potential for the use of the rAAV vector delivered anti-PrP scFvs in prion diseases.

Another method of delivery for scFv is through scFv expressing cells. Donofrio *et al.* produced RD-4 rhabdomyosarcoma cells expressing anti-PrP 6H4 scFv [25]. Filesil *et al.* generated PC12 cells that express a secreted version of the 8H4 anti-prion scFv [26]. In both studies, the secreted anti-PrP scFv inhibited PrP^sc^ formation. More recently, Fujita and colleagues established a Ra2 microglial cell line expressing anti-PrP 3S9scFv. Microglia are known to infiltrate the prion lesions. Delivery of the anti-PrP scFv by Ra2 microglial cells showed a small but significant increase in survival time when the microglial cells were injected into mice seven weeks after 22L scrapie prion infection [43].

A humanized scFv was developed based on the V5B2 anti-PrP^sc^ IgG1 monoclonal antibody (mAb), which recognizes a synthetic peptide representing the C-terminus of the human PrP. The humanized V5B2 scFv was engineered with the aid of computer modeling. The resulting scFv had human amino acid residues and 13 mutations introduced at key positions compared with the original murine mAb, yet retained V5B2 mAb’s stability, specificity and affinity [42,45]. However, the effectiveness of V5B2 scFv has not been evaluated.

Most of the scFv antibodies established so far are either against PrP^c^ or both PrP^sc^ and PrP^c^, and are not conformation specific. The therapeutic potential of the mAbs or scFvs against different PrP conformations were first demonstrated by Petsch *et al.* [44]. In this study, a mAb specific to PrP^sc^ (W261) and a conformation independent mAb against PrP (W226) were used to treat ScN2a cells. Only the conformation independent mAb showed a therapeutic benefit in the ScN2a model. This result agrees with the group’s earlier finding that the complementarity determining regions of W226, which specifically recognize PrP^sc^, did not have anti-prion activity [40]. However, mAb W226 or scFv W226 only showed marginal efficacy *in vivo* when they were used to treat scrapie-infected mice. This finding is inconsistent with an earlier study [51]. A recent study by Kubota *et al.* generated antibodies against the β-rich prion protein [47]. In this study, the conformation-defined recombinant PrP was generated and two human IgGs were established by screening a human scFv phage display library. These two antibodies recognize specifically the β-form but not the α-form of the recombinant PrP of human, bovine, sheep, and mouse. Using this antibody, the authors demonstrated that the β-form specific antibody could not inhibit the accumulation of PrP^sc^, and could only trigger weak apoptosis of prion-infected cells. On the other hand, an anti-PrP antibody recognizing all conformations could inhibit the accumulation of PrP^sc^ and induce apoptotic cell death. In summary, these studies suggest that PrP^c^, instead of PrP^sc^, is the therapeutic target for prion diseases.

An alternative target for prion disease is the 37/67 kDa laminin receptor (LRP/LR), which is believed to act as a receptor for PrP^c^ and PrP^sc^ [52,53]. LRP/LR plays a vital role in prion infection [54,55]. It has been demonstrated to be responsible for bovine PrP^sc^ internalization by human cells [56]. Expression of scFv against LRP/LR in scrappie-infected mice showed reduction of peripheral PrP^sc^ levels but did not prolong survival [57,58].

## 3. scFv Therapy in Alzheimer’s Diseases

AD is the most common neurodegenerative disease affecting more than 36.5 million people worldwide [59,60]. The pathological features of AD include progressive neuronal loss, the accumulation of the Tau protein neurofibrillary tangles, and the formation of amyloid beta (Aβ) plaques. Passive immunotherapy has been demonstrated as the most promising therapeutics method to treat AD [21,61]. Currently efforts are primarily focused on the 4-kDa peptide Aβ, a major pathological peptide contributor to AD. Antibodies has been raised against the *N*-terminal, middle, and *C*-terminal of the monomeric Aβ as well as the soluble Aβ—containing oligomers, protofibrils and fibrils (reviewed in Robert *et al*., 2012 [21]). Like anti-prion antibodies, these antibodies are screened for their ability to prevent Aβ fibril formation or to disrupt existing fibrils. Many of those mAbs have entered clinical trials. Thus far, all clinical trials with these antibodies have failed to demonstrate efficacy.

As mentioned earlier, mAbs as a potential therapeutic reagent also suffer from disadvantages with regard to production, low tissue penetration, and potential serious adverse effects such as mass inflammatory reactions and cerebral amyloid angiopathy associated microhemorrhage [62,63]. These drawbacks may be improved through antibody engineering via the production of scFvs.

The first anti-Aβ scFv was produced by Frenkel *et al.* based on the variable regions of an anti-Aβ IgM 508 antibody [64]. This scFv, named 508F(Fv), could lead to the disaggregation of Aβ fibrils and prevent toxic effects in cultured PC-12 cells. By screening a naïve human scFv phage library with Aβ1–28 and Aβ1–40, Liu *et al.* selected two positive scFv clones, H1v2 and C1, which bound to the *N*-terminal or *C*-terminal of Aβ, respectively [65]. Liu *et al.* also showed that H1v2 but not C1 could inhibit Aβ aggregation *in vitro* [65]. Likewise, engineered scFv antibody based on mAb WO-2, which recognizes Aβ2–8 could inhibit Aβ fibril formation, disaggregate Aβ fibrils, and reduce Aβ oligomer toxicity [61].

Anti-Aβ scFvs can be easily delivered with AAV vectors. Tg2576 mouse AD model injected with rAAV2 expressing anti-Aβ scFv had detectable scFv in the hippocampal neurons after one year of injection. The rAAV2-CAscFv59 injected mice showed lower levels of amyloid deposits without obvious neurotoxicity [66]. Another study with AAV1 delivered anti-Aβ-16, Aβ40, and Aβ42 achieved widespread scFv expression in the mouse brain. The scFvs were cloned from established hybridomas mAb9, mAb40, and mAb42.2, respectively. Intracranial delivery of AAV1 expressing anti-Aβ scFvs did not show any adverse effect in CRND8 mice. About 20%–50% reduction in amyloid deposits were achieved with AAV1-scFv-Aβ in the CRND8 AD mouse model [67]. Wang *et al.* demonstrated that intramuscular delivery of rAAV2-scFv against Aβ was also safe and effectively reduced total Aβ levels in the brain [68].

**Figure 4:**
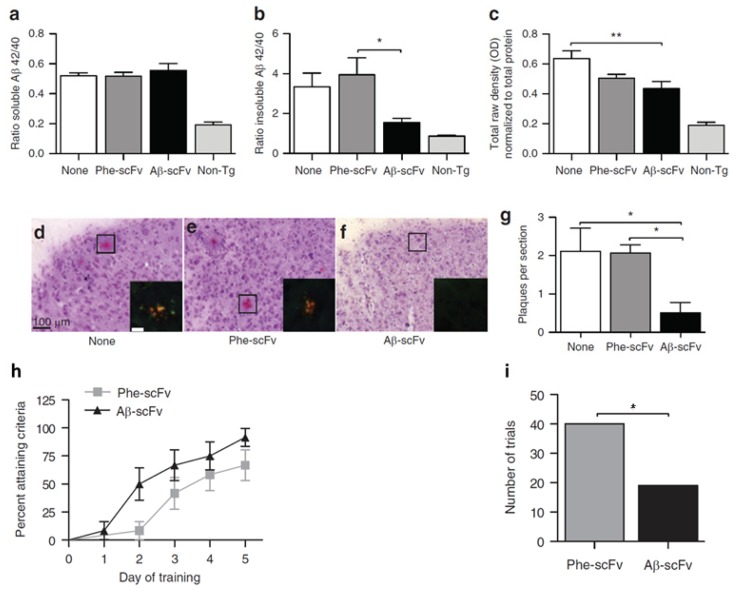
Assessment of recombinant adeno-associated virus (rAAV) delivered single-chain fragment variable (scFv) therapy for Alzheimer’s disease (AD) in 3xTg AD mouse model. Hippocampal injections of rAAV1-Phe-scFv (Phe-scFv), rAAV-Aβ-scFv (Aβ-scFv), or no injection control (None) into three-month-old 3xTg-AD mice led to no change in hippocampal soluble Aβ. (**a**) Levels but significant decreases in insoluble Aβ; (**b**) Levels in the Aβ-scFv group; (**c**) The levels of soluble oligomeric Aβ in the Aβ-scFv treated mice was significantly decreased compared to mice with no injection; (**d**,**e**,**f**) Congo red staining showed reduction of amyloid plaques in Aβ-scFv injected mice; (**g**) Quantitative measurements of amyloid plagues. (*p < 0.05; **p < 0.01; One-way ANOVA with Bonferroni’s multiple comparison *post-hoc* test.); (**h**) Morris Water Maze test showed improved spatial learning in Aβ-scFv treated mice when compared to the Phe-scFv treated mice; (**i**) The total number of trials that Phe-scFv injected mice failed to meet the learning criteria was significantly higher than that of the Aβ-scFv injected mice (*p* = 0.0041, Fisher’s exact test). Figures reproduced from Ryan *et al*., 2010 [69].

We previously showed that intrahippocampally delivered rAAV1-Aβ scFv was able to reduce Aβ and hyperphosphorylated tau levels and improve cognitive functions in a 3xTg-AD mouse model [69]. Compared to no injection control and Phe-scFv control, rAAV expressed anti-Aβ scFv significantly reduced insoluble Aβ and soluble oligomeric Aβ levels in mouse hippocampus, although the soluble Aβ levels remained unchanged (Figure 4). In addition, a > 75% decrease in Congo red-stained amyloid plaques was observed in Aβ-scFv treated 3xTg-AD mice when compared to the controls (Figure 4). Importantly, Aβ-scFv treatment improved spatial learning and memory abilities of the 3xTg-AD mice. In the Morris Water Maze test, 3xTg-AD mice with hippocampal injection of Aβ-scFv learned more quickly than the Phe-scFv controls (Figure 4). In this preclinical study, we validated rAAV-delivered anti-Aβ scFv as a potential therapeutic strategy to attenuate pathologic protein levels and ameliorate behavior defects.

All above studies demonstrated the safety and efficacy of AAV delivered long-term expression of anti-Aβ scFvs as a potentially viable AD treatment strategy.

Four conformation specific anti-Aβ scFvs have been identified from a naïve human scFv phage display library [70]. The four scFvs W8, W9, W20, and WC2 only bind to Aβ oligomers but not the other conformations. All four scFvs bind to the same region on the Aβ oligomer and can inhibit Aβ fibril formation and neurotoxicity [70]. Interestingly, scFvs W8 and W20 not only could recognize Aβ oligomers, but they could also recognize oligomers of other amyloid proteins such as SNCA and PrP. W8 and W20 could inhibit protein aggregation and reduce cytotoxicity of the amyloid protein oligomers [46].

## 4. scFv Therapy in Huntington’s Diseases

Huntington’s Disease (HD) is an autosomal dominant neurodegenerative disorder caused by the expansion of the polyglutamine (polyQ) tract in the Huntington (Htt) protein [71,72]. The expanded *N*-terminal of the Htt protein leads to pathological aggregation of the Htt protein in the patient brain and causes fatal motor disorder. Generally, patients carrying an htt gene with 35–39 polyQ repeats show late onset HD with incomplete penetrance. Patients carrying an htt gene with more than 40 polyQ repeats show early onset HD with 100% penetrance [20,73]. In the past decade, various intracellular anti-Htt immunotherapies have been developed as potential treatment options for HD [20], including anti-Htt scFvs.

By screening the human phage display library, a scFv (C4) recognizing the 17 *N*-terminal Htt was identified by Lecerf *et al.* [74]. Co-expression of this C4 scFv and the expanded repeat Htt-polyQ-GFP reduced the number of Htt aggregates in COS-7 cells [74]. In organotypic slice cultures, co-transfection of C4 scFv could reduce the malonate-induced cell death in mutant Htt-expressing cells [75]. Further study showed that C4 scFv specifically targets the soluble portion of the mutant Htt *N*-terminal fragment and therefore may shift the equilibrium of mutant monomeric *vs.* higher order Htt aggregates [76]. The C4 scFv had been tested *in vivo* with a *Drosophila* HD model that expresses Htt exon 1-Q93. Compared to only 30% of the transgenic flies surviving to adulthood, 100% of flies expressing elav-Gal4 driven UAS-C4 sFv expression reached adulthood. The C4 sFv expression also reduced neurodegeneration and prolonged the lifespan of the HD flies [77,78]. Striatum delivery of AAV2/1 expressing C4 scFv in B6.HDR6/1 mice reduced mHtt aggregates in neurons. Striatal expression of C4 scFv seemed to be well tolerated by mice and did not elicit adverse effects [79]. However, the effectiveness of the AAV delivered C4 scFv decreases over time and age of the animals. In an effort to improve the efficiency of C4 scFv, Butler and Messer generated a scFv fusion protein with a PEST domain. The C4 scFv-PEST fusion protein retained the anti-mutant-Htt function and has increased proteasomal degradation of the mutant Htt [80]. The above studies have established preclinical efficacy of C4 scFv for mutant Htt *in vitro* and *in vivo*.

Khoshnan and colleagues produced scFvs based on mAbs that recognize the polyQ (MW1, MW2) and the polyP (MW7) domains of the Htt exon 1. All three scFvs were found to bind to Htt in 293 cells expressing Htt exon 1 with 103 polyQ repeats. Interestingly, scFvs binding to the polyQ domain of Htt triggered pronounced cell death and Htt aggregation, whereas scFv binding to the polyP domain of Htt inhibited Htt aggregation and cytotoxicity [81]. These results suggest a similar scenario with regard to other amyloid proteins where the region of scFv binding can exhibit differential effects on protein aggregation and related toxicity.

## 5. scFv Therapy in Parkinson’s Diseases

Parkinson’s disease (PD) is a progressive neurodegenerative disordered caused by the loss of nigrostriatal dopaminergic neurons [82,83]. PD is a complex neuronal disease that involves both genetic and environmental factors [84,85]. Defects in many genes have been implemented in the pathogenesis of PD. Among these, SNCA is strongly associated with PD [86–89]. A hallmark of PD is the formation of aggregated SNCA-containing Lewy bodies in dopamine neurons located in the substantia nigra [88,89]. Therefore SNCA has become a major target for PD immunotherapy.

Our laboratory generated conformation-specific humanized scFvs against SNCA. These scFvs can be efficiently expressed in mammalian cells through transductions with HSV vector carrying the scFvs expression cassette [90]. Emadi *et al.* identified 10 anti-SNCA scFvs by screening naïve human phage display libraries. A strong scFv recognizing the *C*-terminal of SNCA could inhibit SNCA aggregation *in vitro* [91]. Co-expressing of anti-monomeric SNCA scFv and SNCA in mammalian cells prevented SNCA aggregation and rescued cellular toxicity caused by SNCA overexpression [92,93]. Similarly, scFv against oligomeric SNCA could also prevent SNCA aggregation and rescue SNCA-mediated cytotoxicity [94].

Although the use of scFv as a therapeutic intervention has been largely overlooked due to the complexity of human Parkinsonism and the uncertainty of disease pathogenesis, these studies provide an encouraging outlook that warrants further study of anti-SNCA scFv in PD models.

## 6. Improvements of scFv for Neuronal Disorder

Many efforts were directed towards improving the performance of the scFv in neurodegenerative diseases; these efforts include reduction of immunogenicity, enhanced scFv stability and solubility, improved avidity, and increased crossing of the blood-brain barrier (BBB). scFvs can be engineered into multimers such as diabodies or triabodies to increase antigen binding [22,95]. scFvs sequence optimization including the replacement of unpaired cysteine residue in the scFv fragment [64], coding region mutagenesis, computer-guided codon humanization [42,45], and addition of the PEST domain to the scFvs [80,96] has helped to increase scFv stability and decrease immune response against the scFvs. In certain cases, changes in scFvs may lead to reduced affinity against cognate antigens. Similarly, fusion protein strategies have been used to target scFvs to specific subcellular locations to enhance interdiction of pathogenic protein [29,96]. Many scFvs exhibit solubility issue when expressed in the cells. Linker length and amino acids composition is known to affect scFv solubility. This can be improved by the addition of charged residues to the linker sequence [97]. In addition, high-throughput selection strategy [98], peptide tag fusion strategy [99], and combination strategy with fusion tags and solubility-enhancing reagents have been developed to increase scFv solubility [100].

Although some scFv are believed to be able to cross the BBB, other scFvs failed in this regard. Two techniques have been developed to help scFv to cross the BBB. One takes advantage of the human insulin receptor (HIR). Boado and colleagues made a fusion protein of anti-Aβ scFv with a HIR mAb. The resulting fusion protein can be transported into the Rhesus monkey brain through binding to HIR [101]. Another approach uses the cell-penetrating peptide (CPP). In this case, the CPP replaced the linker peptide of scFv and the resulting scFv-CPP fusion protein was effective in crossing the BBB in mice [102].

Moreover, more appropriate disease targeting scFvs can be generated based on available mAb panels or natural occurring autoimmune antibodies that bind to aggregated proteins. A comprehensive collection of anti-PrP^c^ mAb has been developed against different epitopes located across the entire mouse PrP^c^ protein [38]. Similar comprehensive mAb panels can be developed as diagnostics and candidate therapeutics. It is also possible to engineer scFvs based on natural occurring antibodies directed against amyloid proteins. Human autoantibodies have been shown to inhibit PrP aggregation and relieve neurotoxicity [22,103].

## 7. Right Target at the Right Place

Amyloid proteins exist in different conformations. In most cases, monomer, oligomer, protofibril, and fibril forms of the amyloid proteins coexist. In recent years, more evidence has shown that neurotoxicity is caused primarily by the monomeric and/or oligomeric forms of the disease-associated amyloid proteins; the formation of the aggregates is likely to be a protective process [2,104–106]. For example, SNCA fibrils are mostly present in Lewy bodies and are considered as structurally stable with low toxicity [89]. In contrast, oligomer forms of SNCA have been detected in diseased brain and believed to change neuronal membrane permeability [107,108], damage mitochondria [109], disrupt microtubules [110] (reviewed by Lashuel *et al.* and Brown [106,111]). The monomeric or oligomeric forms of PrP have been implicated in neurotoxicity *in vitro* and *in vivo* [112–115]. In addition, much evidence supports that Aβ oligomers are neurotoxic (reviewed by Gilbert *et al.* [2]).

Although the debate over whether the oligomer or the fibril aggregate is not yet settled, we believe scFvs targeting the monomeric and oligomeric forms represent the most important therapeutic opportunities for AD, PD, HD, and prion diseases. Removal of soluble amyloid proteins eliminates the substrates for toxic oligomer/protofibril formation. This is clearly demonstrated in Prnp^0/0^ mice, which are resistant to prion infections [116]. scFvs can also change the conversion dynamics between different forms of these proteins. By removing the monomeric and oligomeric forms of the amyloid proteins, scFvs change the equilibrium, disaggregate proteins and potentially reduce neurotoxicity. Indeed, as we have summarized in the previous sections, scFvs against PrP, Aβ, SNCA monomers or oligomers have demonstrated neuroprotection [40,43,46,47,61,70,91,93,94,117]. Conformation specific scFvs against the fibril forms of the proteins are not effective in reducing protein aggregates and neurotoxicity [33,42,44,47,81].

**Figure 5:**
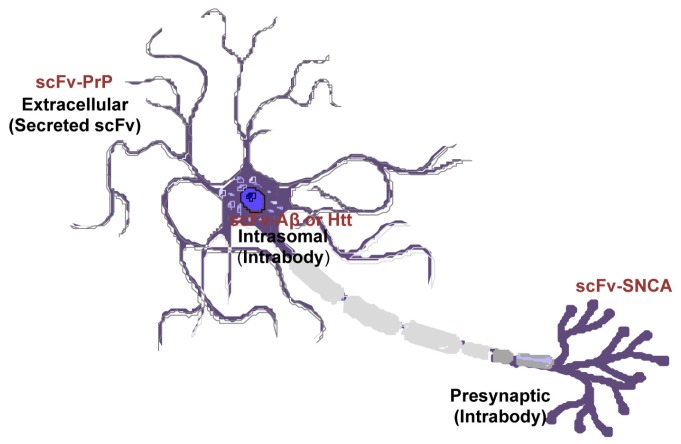
Differential cellular location of single-chain fragment variable (scFv) that targets specific pathogenic antigen in different neurodegenerative diseases. Recombinant adeno-associated virus (rAAV)-scFv can be engineered to localized to desired subcellular regions (intrabodies), or as a secreted peptide to target membrane-bound or extracellular proteins.

Amyloid proteins are located at a variety of locations: PrP has an extracelluar domain; Aβ is both intracellular and extracellular; SNCA is present on the membrane and within cells; Htt is mostly cytoplasmic. However, the subcellular location of native proteins and their pathological forms can be different. SNCA fibrils are mostly resided in the neuronal cell body whereas the oligomeric forms are predominantly located at the axon and synapses [118–121]. Mutant Htt in diseased neurons changes its localization from cytoplasm to the nucleus [122]. The scFvs can be engineered to localize to desired subcellular regions (intrabodies), or configured as a secreted molecule to engage membrane-bound or extracellular targeted proteins (Figure 5). Previous studies have found endoplasmic reticulum-targeted [29], secreted [25,26,43], or intracellular expressed [81] scFvs are effective in disaggregate amyloids depending on the types of the proteins or the pathological state. Future work is needed to elucidate the subcellular locations where amyloid proteins produce greatest toxicity. With this information, more targeted and effective therapeutics are envisaged.

## 8. Conclusions

As reviewed above, the scFvs have been developed as a passive immunotherapy and evaluated in models of neurodegenerative disorders caused by pathogenic misfolded proteins. This potential therapeutic has unique advantages although additional work on scFv-based therapy is required to improve solubility, tissue retention, rapid turnover, lower avidity, scFv-antigen complex clearance from brain, and potential adverse effects due to the targeting of normal functions of self-antigens. Careful toxicity study is required for any scFv prior to its clinical use. Accordingly, it is prudent to refine scFvs therapeutic action in animal models and judiciously extend a candidate in clinical trials in a neurodegenerative disease where unmet medical need is great.
